# Using Segment Anything Model 2 for Zero-Shot 3D Segmentation of Abdominal Organs in Computed Tomography Scans to Adapt Video Tracking Capabilities for 3D Medical Imaging: Algorithm Development and Validation

**DOI:** 10.2196/72109

**Published:** 2025-04-29

**Authors:** Yosuke Yamagishi, Shouhei Hanaoka, Tomohiro Kikuchi, Takahiro Nakao, Yuta Nakamura, Yukihiro Nomura, Soichiro Miki, Takeharu Yoshikawa, Osamu Abe

**Affiliations:** 1 Division of Radiology and Biomedical Engineering, Graduate School of Medicine, The University of Tokyo, 7-3-1 Hongo, Bunkyo-ku, Tokyo, 113-8655, Japan, 81 3-3815-5411; 2 Department of Radiology, The University of Tokyo Hospital, Tokyo, Japan; 3 Department of Radiology, School of Medicine, Jichi Medical University, Shimotsuke, Japan; 4 Department of Computational Diagnostic Radiology and Preventive Medicine, The University of Tokyo Hospital, Tokyo, Japan; 5 Center for Frontier Medical Engineering, Chiba University, Chiba, Japan

**Keywords:** artificial intelligence, medical image processing, computed tomography, abdominal imaging, segmentation, AI

## Abstract

**Background:**

Medical image segmentation is crucial for diagnosis and treatment planning in radiology, but it traditionally requires extensive manual effort and specialized training data. With its novel video tracking capabilities, the Segment Anything Model 2 (SAM 2) presents a potential solution for automated 3D medical image segmentation without the need for domain-specific training. However, its effectiveness in medical applications, particularly in abdominal computed tomography (CT) imaging remains unexplored.

**Objective:**

The aim of this study was to evaluate the zero-shot performance of SAM 2 in 3D segmentation of abdominal organs in CT scans and to investigate the effects of prompt settings on segmentation results.

**Methods:**

In this retrospective study, we used a subset of the TotalSegmentator CT dataset from eight institutions to assess SAM 2’s ability to segment eight abdominal organs. Segmentation was initiated from three different z-coordinate levels (caudal, mid, and cranial levels) of each organ. Performance was measured using the dice similarity coefficient (DSC). We also analyzed the impact of “negative prompts,” which explicitly exclude certain regions from the segmentation process, on accuracy.

**Results:**

A total of 123 patients (mean age 60.7, SD 15.5 years; 63 men, 60 women) were evaluated. As a zero-shot approach, larger organs with clear boundaries demonstrated high segmentation performance, with mean DSCs as follows: liver, 0.821 (SD 0.192); right kidney, 0.862 (SD 0.212); left kidney, 0.870 (SD 0.154); and spleen, 0.891 (SD 0.131). Smaller organs showed lower performance: gallbladder, 0.531 (SD 0.291); pancreas, 0.361 (SD 0.197); and adrenal glands—right, 0.203 (SD 0.222) and left, 0.308 (SD 0.234). The initial slice for segmentation and the use of negative prompts significantly influenced the results. By removing negative prompts from the input, the DSCs significantly decreased for six organs.

**Conclusions:**

SAM 2 demonstrated promising zero-shot performance in segmenting certain abdominal organs in CT scans, particularly larger organs. Performance was significantly influenced by input negative prompts and initial slice selection, highlighting the importance of optimizing these factors.

## Introduction

Medical image segmentation is a critical task in radiology, playing a vital role in diagnosis, treatment planning, and clinical research [1,2]. Traditionally, this process has been labor-intensive, requiring manual delineation by skilled radiologists. However, recent advancements in deep learning have revolutionized this field, expanding the scope of automated analysis and significantly enhancing performance across diverse medical imaging tasks.

The Segment Anything Model (SAM), introduced by Meta AI, represented a significant leap forward in image segmentation technology [3]. Trained on over a billion masks, SAM demonstrated remarkable versatility in segmenting a wide array of objects across various domains. SAM’s zero-shot performance—its ability to segment objects it has never seen during training—in medical images has been extensively evaluated [4,5], and specialized models such as MedSAM [6], which underwent additional training for medical imaging applications, have been introduced. These developments have demonstrated SAM’s potential in radiological domains, including CT and magnetic resonance imaging (MRI). However, SAM was primarily designed for 2D image segmentation, which imposed inherent limitations on its direct applicability to 3D volumetric data.

SAM 2, released in July 2024, introduced video segmentation capabilities [7], applicable to 3D medical imaging like CT scans. Although not specifically designed for medical use, its zero-shot ability and video tracking features offer a promising approach to 3D medical image segmentation, potentially overcoming limitations of traditional methods that require extensive domain-specific training. Testing SAM 2’s zero-shot performance is crucial because it could significantly reduce the need for large, annotated medical datasets and specialized model training, potentially accelerating the deployment of artificial intelligence in various medical imaging applications.

SAM 2’s zero-shot performance in radiology and the impact of input factors remain understudied, despite evaluations in surgical video segmentation [8] and specialized versions like Medical SAM2 [9]. We assess SAM 2’s zero-shot performance in medical imaging, examining how target organ size, initial slice selection, and negative prompts influence its segmentation accuracy. These factors are crucial for optimizing SAM 2’s performance in radiological applications.

We focused our evaluation on abdominal organs due to their significant clinical importance. Morphological and size analysis of these organs is crucial for disease detection [10]; pancreatic atrophy may indicate highly fatal pancreatic cancer [11] and liver morphology changes can signal cirrhosis [12]. Renal atrophy is associated with chronic kidney diseases [13], and a recent study has shown that kidney volume measurements obtained through accurate segmentation models effectively predict kidney function [14]. These volumetric assessments require precise 3D segmentation, making abdominal imaging an ideal test case for evaluating SAM 2’s capabilities in clinically relevant scenarios.

This comprehensive evaluation combining zero-shot performance assessment and input factor analysis is one of the earliest investigations for SAM 2 applied to 3D medical imaging. Our exploration is analogous to large language models, where performance varies significantly based on prompt adjustments [15]. By examining prompt engineering in segmentation, we aim to provide deeper insights into adapting general-purpose AI models for specialized medical imaging applications.

## Methods

This study was conducted as a retrospective study and adheres to the Checklist for Artificial Intelligence in Medical Imaging (CLAIM): 2024 Update [16,17].

### Ethical Considerations

We used the openly available TotalSegmentator dataset [18], a CT image segmentation dataset. The TotalSegmentator dataset is released under the Creative Commons Attribution 4.0 International license, permitting unrestricted reuse for research. The original CT images were collected retrospectively at University Hospital Basel. The Ethics Committee Northwest and Central Switzerland (EKNZ) approved a waiver of ethical approval for that retrospective study (BASEC Req-2022–00495). No additional ethics review was required for this secondary analysis. Patient consent was waived by the EKNZ due to the deidentified, retrospective nature of the original data collection. All CT images in the TotalSegmentator dataset were fully deidentified of any protected health information before public release. The dataset contains anonymized images and no patient identifiers. No financial or other compensation was provided to participants for the original data collection, and none was provided for this secondary analysis.

### Dataset

We aimed to evaluate the segmentation performance of major organs within the imaging range of abdominal CT, one of the most common medical imaging modalities. To conduct this performance evaluation, we used a subset of the TotalSegmentator CT dataset version 1.0 [18]. The TotalSegmentator dataset is a large-scale, multiorgan segmentation dataset collected from eight institutions. We selected this dataset for its comprehensive organ segmentation masks and available institutional metadata for each case. These segmentation masks underwent expert verification to ensure high quality, making the dataset particularly suitable for our study.

Our study included cases that encompassed the abdominal region, while CT angiography scans were excluded from the analysis.

To ensure representation from all eight institutions while managing the dataset size, we implemented a sampling strategy. We set a maximum of 20 cases per institution and randomly selected cases up to this limit. For institutions with fewer than 20 cases, all available cases were included.

We focused on 8 major abdominal organs for our analysis:

1. Liver
2. Right kidney
3. Left kidney
4. Spleen
5. Gallbladder
6. Pancreas
7. Right adrenal gland
8. Left adrenal gland

**Figure 1.**
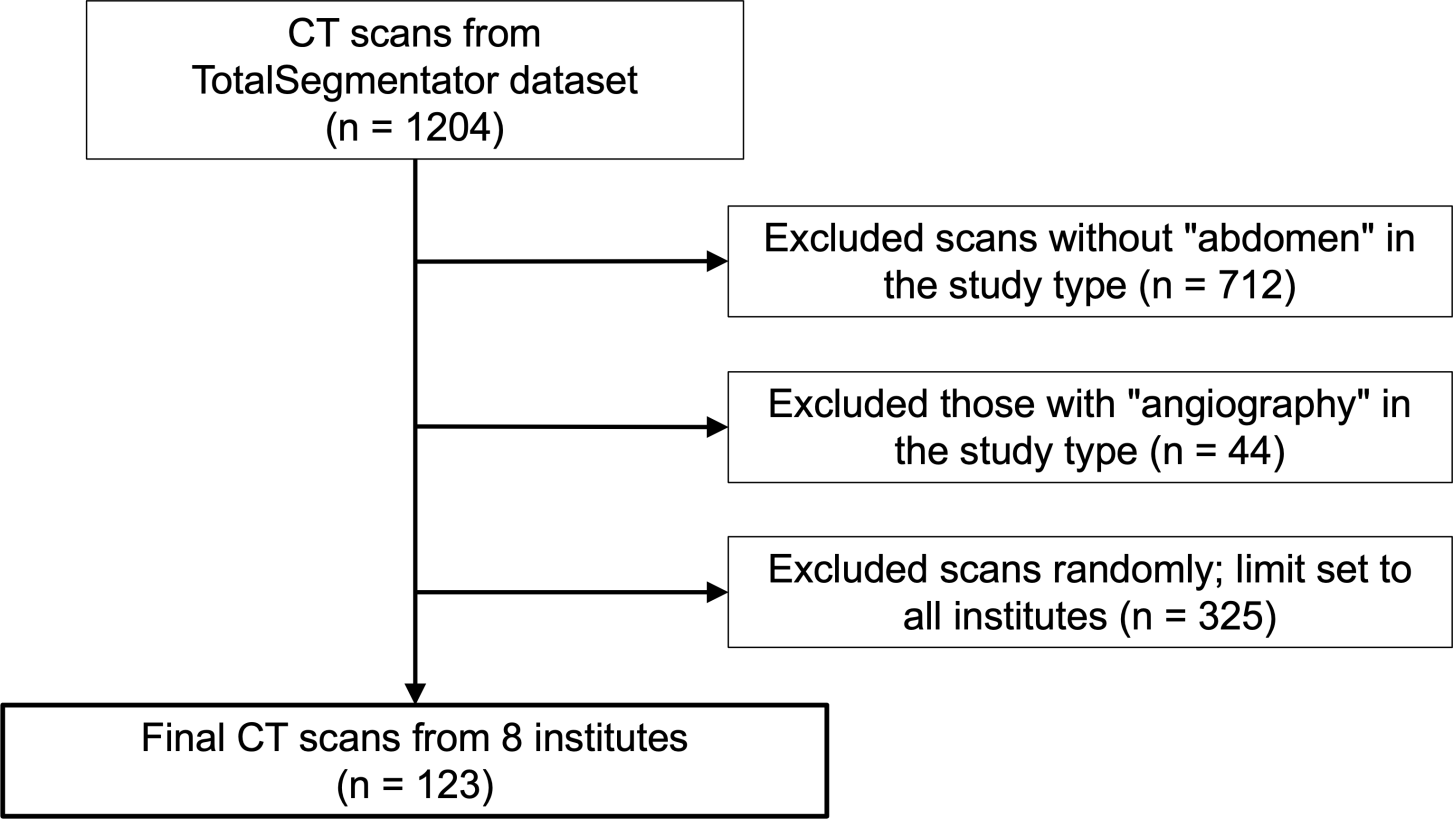
Flow diagram illustrating the CT scan selection process from the TotalSegmentator dataset for evaluation of SAM 2. CT: computed tomography; SAM 2: Segment Anything Model 2.

These organs were selected based on their clinical significance and visibility in standard abdominal CT scans. To account for potential annotation deficiencies, we excluded segmentation masks with extremely small volumes by setting a threshold of 100 voxels. Masks below this threshold were omitted from the analysis. The dataset selection flowchart is illustrated in Figure 1.

### Data Preprocessing

The dataset was available in NIfTI file format. For SAM 2 inference, we extracted each horizontal slice from the 3D volumes to create subsets of 2D images for each scan. We applied windowing to the CT scans, using a window level of 50 and a window width of 400 Hounsfield units. Following windowing, we performed min-max scaling on the data. The scaled values were then converted to 8-bit integers, resulting in a range of 0‐255. These processed 2D images were saved as sequential JPEG files.

For SAM 2 inference, we selectively processed only the slices containing abdominal organs. This approach focused on optimizing computational efficiency, resulting in faster inference speeds.

### Analysis of Organ Mask Volumes

For the volumetric analysis, we used the existing segmentation masks from the TotalSegmentator dataset to calculate the volume of each organ in voxels. We chose to measure in voxels rather than physical units, as our model inputs do not consider voxel scale.

Additionally, we analyzed cross-sectional areas of organ masks along the z-axis. For each organ, we calculated mean mask areas at the 25th, 50th, and 75th percentile z-coordinates, corresponding to the initial prompt locations used in SAM 2 segmentation.

### SAM 2 Implementation for 3D Medical Image Segmentation

**Figure 2.**
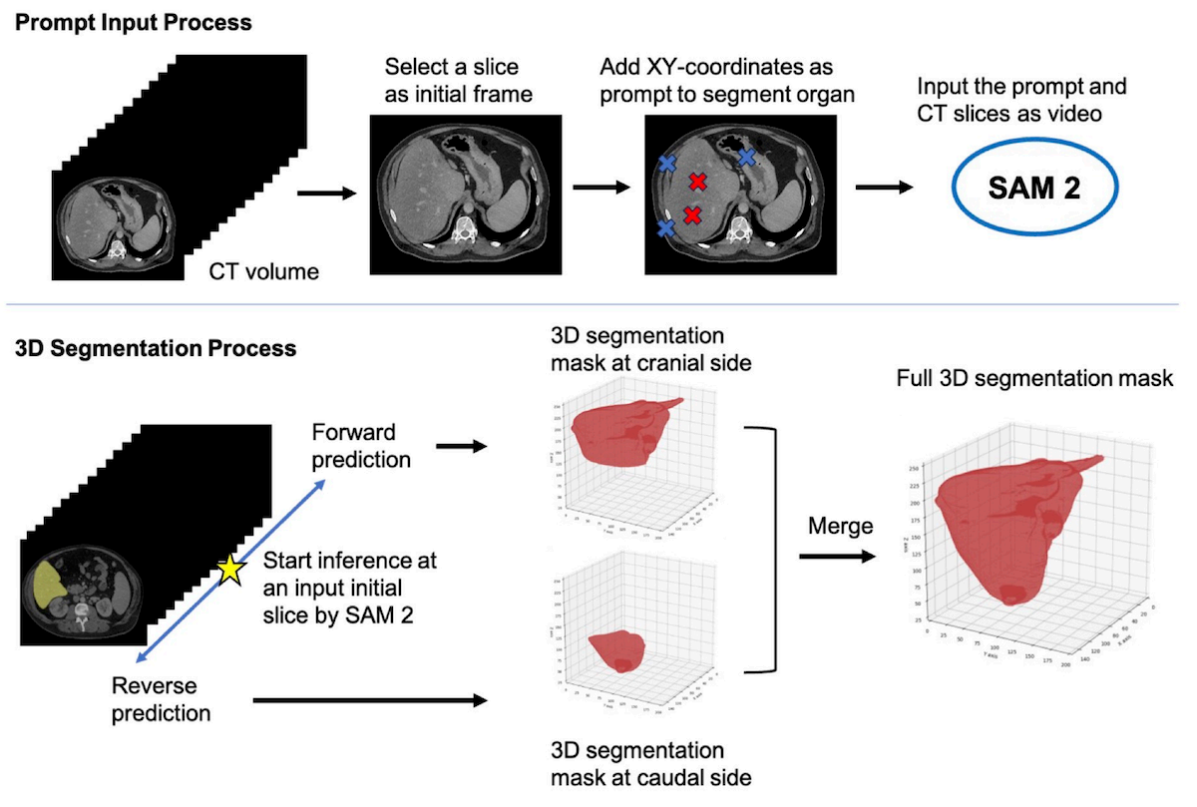
Workflow of 3D medical image segmentation using SAM 2. This figure illustrates a two-stage process for 3D medical image segmentation. The top row shows the prompt input process, where a slice is selected from a CT volume as an initial frame and XY-coordinates are added as prompts for organ segmentation (red crosses: positive prompts; blue crosses: negative prompts). These are then input into SAM 2. The bottom row depicts the 3D segmentation process, where SAM 2 performs forward and reverse predictions to generate 3D segmentation masks at both cranial and caudal sides. These masks are ultimately merged to create a full 3D segmentation mask of the target organ. CT: computed tomography; SAM 2: Segment Anything Model 2.

SAM 2 is a segmentation model not specifically designed for medical images, but for general video content such as sports or animal footage. These models are trained on a large-scale dataset, enabling them to perform segmentation on any object. SAM 2’s main feature is its ability to support not only 2D images but also videos. By providing coordinates indicating the target object for segmentation, SAM 2 can track and segment objects appearing within the video. An overview of CT volume segmentation using SAM 2’s video predictor is shown in Figure 2.

#### Adaptation for 3D Medical Imaging

Although SAM 2 was originally intended for tracking objects in general videos, we recognized that 3D volumes such as CT and MRI scans can be considered as videos composed of numerous 2D images. Using publicly available datasets with segmentation masks, we applied preprocessing compatible with SAM 2’s video prediction capabilities. This allowed us to construct a pipeline capable of performing multiorgan segmentation in a zero-shot manner, without additional training of SAM 2.

#### Bidirectional Prediction Approach

While SAM 2’s video prediction is unidirectional, it can process in both directions from the initial frame. To obtain a complete segmentation mask for the entire volume, we implemented a simple bidirectional approach: forward direction from the starting slice to the cranial end reverse direction from the starting slice to the caudal end. The two segmentation masks obtained from these bidirectional inferences were then merged to create a complete 3D segmentation mask.

#### Model Inference and Prompt Setting

SAM 2’s video prediction requires input of both the video (numbered 2D images) and prompt (coordinates for the target object). We used axial slices for prompt input, as these views serve as the foundation of radiological interpretation in clinical practice. This approach aligns with the standard workflow of radiologists when adapting SAM2 for medical image analysis. In practice, the prompt must be manually specified by a user. However, given the need to evaluate a large number of objects, we devised an algorithm to automatically obtain prompts:

1. Z-coordinate focus: Using 25th (caudal-level), 50th (mid-level), and 75th percentiles (cranial-level) for comprehensive organ representation.
2. Random selection within organ boundaries:
  - Five positive prompts from within the segmentation mask
  - Five negative prompts were sampled from regions 2‐3 voxels outside the mask boundary, excluding the immediate 1-voxel margin

This method maintains reproducibility, reduces bias from the user’s prompting skill, and leverages SAM 2’s capability to use both positive and negative prompts for improved accuracy.

In this study, we refer to the 3D segmentations initiated at each of these positions as caudal-approach, mid-approach, and cranial-approach, corresponding to segmentations starting from the caudal, mid, and cranial-level slices, respectively.

#### Model Version

For our study, we selected the “sam2_hiera_large” model due to its superior performance among the available versions. We used version 1.0 of the SAM 2 [19]. Our implementation was carried out using Python (version 3.10.12).

### Statistical Analysis

To evaluate the model’s performance, we calculated the Dice similarity coefficient (DSC) [20]. This evaluation was performed organ-wise across the dataset to provide a detailed analysis.

We then compared the segmentation performance across the different approaches with or without negative prompts. We performed three pairwise comparisons for the approaches: caudal-approach versus mid-approach, caudal-approach versus cranial-approach, and mid-approach versus cranial-approach. Additionally, we compared performance with and without negative prompts.

When considering organ volumes in detail, we calculated Spearman correlation coefficients to examine the relationship between organ volumes and DSCs [21,22].

To account for multiple comparisons, we applied the Bonferroni correction. After Bonferroni correction, a P value of <.05/3 (approximately 0.0167) was considered statistically significant.

All statistical analyses were conducted using SciPy (version 1.14.0).

## Results

### Data Characteristics

Our sampling strategy resulted in a total of 123 scans. Twenty scans each were selected from five institutions, while the remaining three institutions contributed 5, 5, and 13 cases respectively. The average age of the patients in our selected sample was 60.7 (SD 15.5) years. The gender distribution was nearly equal: 63 male and 60 female individuals.

903 organ segmentations were obtained from 123 scans. 12 masks with volumes of 100 voxels or smaller were then excluded from the analysis, and the final dataset consisted of 891 organ segmentations.

### Analysis of Organ Mask Volumes and Areas

Organ volumes, measured in voxels and are detailed in Table 1. The liver was the largest organ, followed by the spleen; kidneys were next in size, with similar volumes on the left and right side. Compared to the liver’s mean volume, the pancreas was approximately 1/25th the size of the liver; the gallbladder was less than 1/70, and both adrenal glands were less than 1/400. These three organs (ie, pancreas, gallbladder, and adrenal glands) can be categorized as small organs.

**Table 1.** Descriptive statistics of organ volumes in voxels derived from computed tomography scan mask volumes.

Organ	Organ volumes (voxels), mean (SD)	Min^a^	Max^b^	Samples (n)
Liver	465,008.6 (156,091.00)	19,768	963,401	119
Right kidney	39,381.57 (18,122.20)	216	79,713	108
Left kidney	41,246.74 (21,144.60)	666	129,706	111
Spleen	71,730.34 (45,884.40)	13,818	303,676	115
Gallbladder	6,247.61 (4,902.72)	170	20,763	89
Pancreas	18,526.41 (8,502.56)	707	37,855	116
Right adrenal gland	1,101.86 (465.47)	216	2590	118
Left adrenal gland	1,259.03 (522.81)	135	2977	115

a Min: Minimum

b Max: Maximum

Organ cross-sectional area analysis showed diverse trends across eight organs. The liver was the largest in size, increasing caudally to cranially. The pancreas steadily increased while adrenal glands, though smallest, peaked at mid-level. The details are provided in Figure S1 in Multimedia Appendix 1.

### Multiorgan Segmentation Performance

We evaluated the performance for multiorgan segmentation using different starting slice positions. The DSCs are reported as mean (SD) to reflect performance variability. All results are detailed in Table 2.

**Table 2.** DSCs^a^ for multiorgan segmentation by different approaches (ie, caudal, mid, and cranial).

Organ and approach	DSC,^a^ mean (SD)	*P* value^b^
Liver		caudal versus mid: <.01 caudal versus cranial: <.01mid versus cranial: .07
caudal	0.821 (0.192)	
mid	0.754 (0.223)	
cranial	0.702 (0.259)	
Right kidney		caudal versus mid: .03 caudal versus cranial: .16 mid versus cranial: <.01
caudal	0.862 (0.189)	
mid	0.862 (0.212)	
cranial	0.801 (0.270)	
Left kidney		caudal versus mid: .40 caudal versus cranial: .15 mid versus cranial: .15
caudal	0.870 (0.154)	
mid	0.825 (0.221)	
cranial	0.808 (0.242)	
Spleen		caudal versus mid: <.01 caudal versus cranial: .017 mid versus cranial: .56
caudal	0.891 (0.131)	
mid	0.839 (0.187)	
cranial	0.768 (0.302)	
Gallbladder		caudal versus mid: .95 caudal versus cranial: <.01mid versus cranial: .08
caudal	0.527 (0.288)	
mid	0.531 (0.291)	
cranial	0.461 (0.314)	
Pancreas		caudal versus mid: .92 caudal versus cranial: <.01mid versus cranial: <.01
caudal	0.353 (0.168)	
mid	0.361 (0.197)	
cranial	0.287 (0.209)	
Right adrenal gland		caudal versus mid: <.01 caudal versus cranial: <.01mid versus cranial: <.01
caudal	0.203 (0.222)	
mid	0.177 (0.235)	
cranial	0.112 (0.178)	
Left adrenal gland		caudal versus mid: <.01 caudal versus cranial: <.01mid versus cranial: .08
caudal	0.308 (0.234)	
mid	0.252 (0.226)	
cranial	0.226 (0.238)	

a DSC: Dice Similarity Coefficient.

b *P* values from Wilcoxon signed-rank tests are provided for comparisons between approaches (caudal vs mid, caudal vs cranial, and mid vs cranial).

The left kidney demonstrated the best overall performance, maintaining high DSCs across all starting positions: mean 0.870 (SD 0.154) , mean 0.825 (0.221), and 0.808 (SD 0.242) for the caudal-approach, mid-approach, and cranial-approach, respectively. Notably, it was the only organ showing no statistically significant differences between any starting positions (all *P*>.0167).

**Figure 3.**
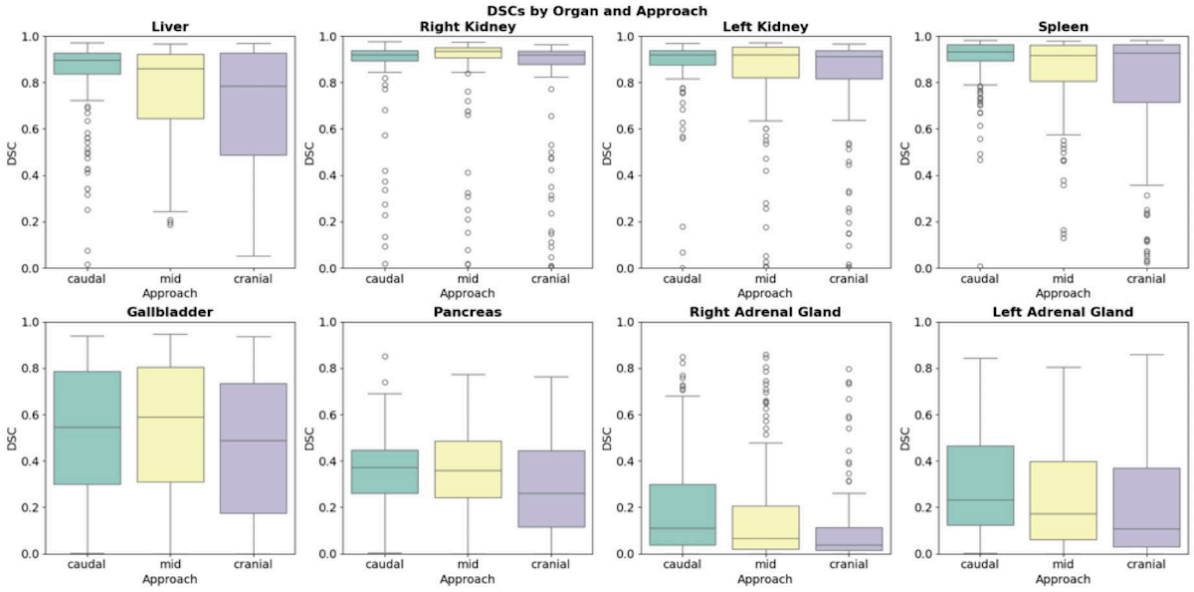
Box plots of DSCs for eight organs (displayed in separate subplots) across three approaches: caudal-approach, mid-approach, and cranial-approach. Each subplot shows the distribution of DSCs (y-axis, range 0‐1) for a specific organ, with the three approaches compared along the x-axis. DSC: Dice Similarity Coefficient.

The box plots (Figure 3) show that most organs reached DSCs above 0.8, with some approaching or nearly reaching 1.0. However, the box plots also reveal instances of very low DSC values approaching 0 across various organs and approaches, indicating significant variability in segmentation performance.

Starting slice level had a significant impact on most organs. Organs demonstrated various patterns in segmentation performance depending on the starting level. The liver showed a significant decrease in performance as the starting position moved superiorly, with DSC dropping from mean 0.821 (SD 0.192) with mean caudal-approach to 0.702 (SD 0.259) with cranial-approach (*P<*.01). Smaller organs such as the pancreas, adrenal glands, and gallbladder showed the most pronounced impact of the starting position. For these organs, performance significantly decreased when changing from a caudal-approach to a cranial-approach (all *Ps<.*01).

Larger organs such as liver, kidneys, and spleen consistently demonstrated higher DSCs compared to smaller organs across all approaches. A moderate correlation was observed across all settings when using caudal-approach (ρ=0.731; *P<.*01), mid-approach (ρ=0.698; *P<.*01), and cranial-approach (ρ=0.699; *P<.*01) (12,13). As shown in Table 3, when correlation coefficients were calculated separately for each organ, fair correlations were demonstrated for almost all items, particularly for smaller organs.

We also investigated the impact of including negative prompts on segmentation performance across different organs, focusing specifically on the caudal-approach (Table 4 and Figure S2 in Multimedia Appendix 1). All organs except the liver (*P*=.32) and spleen (*P*=.27) demonstrated significant increases in DSC (*P<.*01) with the inclusion of negative prompts.

**Table 3.** Spearman correlation coefficients between ground truth values of organ volumes and dice similarity coefficients in caudal, mid, and cranial levels.

Organ	Spearman correlation coefficient, ρ (caudal) ρ^a^	*P* value	Spearman correlation coefficient, ρ (mid)^a^	*P* value	Spearman correlation coefficient, ρ (cranial)^a^	*P* value
Liver	0.328	<.01	0.0489	.60	0.163	.08
Right kidney	0.231	.02	0.347	<.01	0.509	<.01
Left kidney	−0.0107	.91	0.293	<.01	0.295	<.01
Spleen	0.38	<.01	0.307	<.01	0.355	<.01
Gallbladder	0.499	<.01	0.509	<.01	0.469	<.01
Pancreas	0.475	<.01	0.386	<.01	0.379	<.01
Right adrenal gland	0.371	<.01	0.424	<.01	0.278	<.01
Left adrenal gland	0.452	<.01	0.339	<.01	0.447	<.01

a Spearman rank correlation coefficient (ρ) was used to examine the relationship between object volumes and dice similarity coefficients.

**Table 4.** Comparison of multiorgan segmentation performance without negative prompts.

Organ	DSC^a^ mean (SD)	Difference^b^	*P* value^c^
Liver	0.785 (0.244)	−0.036	.32
Right kidney	0.858 (0.203)	−0.004	<.01
Left kidney	0.847 (0.192)	−0.023	<.01
Spleen	0.867 (0.213)	−0.024	.27
Gallbladder	0.438 (0.338)	−0.089	<.01
Pancreas	0.277 (0.197)	−0.076	<.01
Right adrenal gland	0.084 (0.151)	−0.119	<.01
Left adrenal gland	0.190 (0.230)	−0.118	<.01

a DSC: dice similarity coefficient.

b Change when negative prompts are excluded (negative values indicate lower performance without prompts).

c *P* value represents the results of Wilcoxon signed-rank tests comparing performance with, and without negative prompts for each organ.

**Figure 4.**
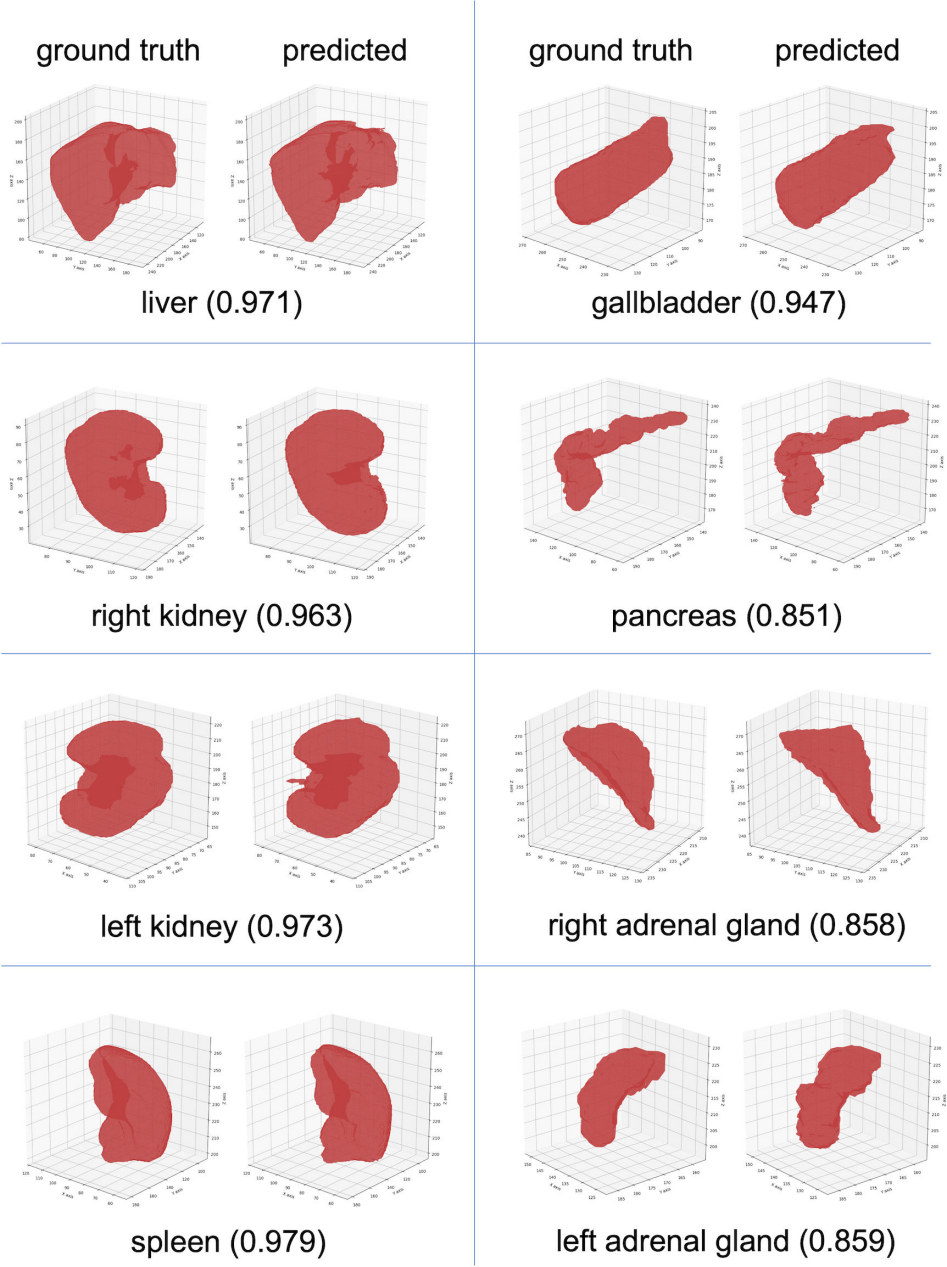
Successful segmentation results for eight abdominal organs. Each row shows different organ with ground truth (left) and predicted (right) 3D masks. Values in parentheses indicate the DSC for each segmentation. DSC: dice similarity coefficient.

In Figure 4, we present the highest DSC masks, excluding cases where the ground truth segmentations were incomplete. The highest performing masks, as visualized, generated for each organ in 3D were nearly indistinguishable from the ground truth.

**Figure 5.**
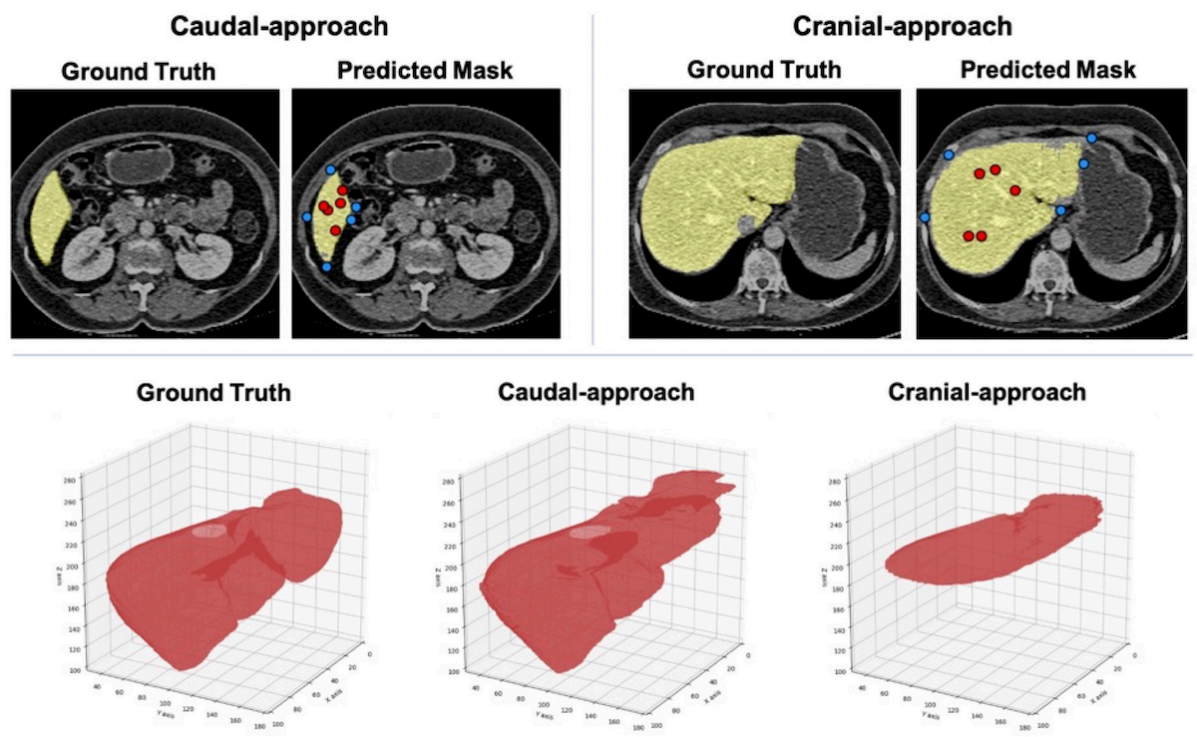
Comparison of segmentation results using caudal-approach and cranial-approach, showing 2D axial slices with ground truth and predicted mask for both initial slices (top row, with yellow representing the ground truth of liver, blue and red points indicating negative and positive prompts, respectively), alongside 3D renderings of liver segmentation for ground truth, caudal-approach, and cranial-approach (bottom row).

On the other hand, there were cases where the performance fluctuated significantly due to differences in the approach. We present an example of the liver segmentation results in Figure 5. The DSC decreased by 0.564 (from 0.924 with caudal-approach to 0.360 with cranial-approach). For the caudal-approach, the initial slice segmentation appears to have been easier due to clear contrast with the surroundings. The cranial-approach resulted in lower DSC values compared to the caudal-approach. Visual inspection of the segmentation results showed incomplete masking and potential inclusion of the inferior vena cava in the liver mask, particularly in areas where boundaries between the liver, inferior vena cava, and abdominal wall were less distinct.

## Discussion

To our knowledge, this is the first research that not only validates the performance of zero-shot SAM 2 on abdominal organs but also considers the impact of prompt input strategies such as slice positioning and negative prompts. Our findings demonstrate the potential of SAM 2, a general-purpose segmentation model in segmenting abdominal organs from CT scans. SAM2 showed promising performance for larger organs with clear boundaries, such as the liver, kidneys, and spleen, achieving a mean DSC of 0.821‐0.891. Although SAM 2 was not specifically designed for medical image analysis, its notable performance suggests potential applicability to a wide range of organs and lesions. The choice of initial prompt position had a significant impact on segmentation accuracy, and the optimal position depended on the organ. Excluding negative prompts led to a significant decrease in DSC for all organs except the spleen and liver, highlighting their importance in segmentation accuracy. SAM 2 struggled with smaller and less defined structures such as the adrenal glands, pancreas, and gallbladder, resulting in lower DSCs. Interestingly, we observed a moderate positive correlation between organ volume and DSCs (ρ=0.731, *P*<.01), suggesting that volume size is one of several key factors influencing segmentation accuracy.

While prior studies have explored zero-shot segmentation performance in CT and MRI [23,24], our research makes several unique contributions to this emerging field. Ma et al [23] conducted a comprehensive benchmarking of SAM 2 across multiple medical image modalities and demonstrated its potential for transfer learning in the medical domain. Similarly, Dong et al [24] explored various prompt strategies and propagation directions for 3D segmentation. In contrast, our work specifically examines abdominal CT imaging, analyzing how prompt positioning and negative prompts significantly influence segmentation outcomes. Unlike previous studies, we investigated segmentation from clinically relevant positions (ie, caudal, mid, and cranial) and found that optimal starting positions vary by organ, with negative prompts being crucial for smaller organ segmentation.

A key advantage of SAM 2 is its ability to generate segmentation masks with just a few clicks on a single slice, drastically reducing the workload for radiologists who previously relied on labor-intensive manual annotations. Furthermore, optimizing prompt input strategies is essential for achieving even greater model performance, as evidenced by SAM’s history of various prompt optimization techniques, including automatic prompt generation and learnable prompts [25]. Although the scores are lower compared to previous supervised methods, which can achieve mean DSCs in the upper 0.9 range for some organs, they are still notably high for a zero-shot prediction. Moreover, the ability to segment an entire 3D volume by simply selecting and clicking on a target structure in a single slice is particularly significant. This aligns with challenges typically observed in abdominal organ segmentation, even with supervised 3D models. Notably, supervised approaches like TotalSegmentator (based on nnUNet [26]), UNet [27], SegUNet [28], and SwinUNETR [29] also tend to show lower DSC for bilateral adrenal glands and gallbladder compared to other organs [30], a trend mirrored in SAM 2’s performance. Segmentation performance can be inferred to depend on multiple factors related to the 3D morphology, volume size, and contrast with surrounding tissues of target structure. These findings suggest the importance of optimizing prompts taking into account the characteristics of the targeted structure.

Our study had several limitations. First, our validation relied on a single dataset of abdominal CT scans, despite being a multi-institutional study. For studies focused on abdominal organs, there are publicly available datasets such as AbdomenCT-1K [31], which is included in AbdomenAtlas [32,33]. To expand the validation to other anatomical structures and imaging modalities, datasets such as Vertebral Segmentation [34], TotalSegmentator’s MRI [35] and Duke Liver datasets [36] could also be considered, all of which include segmentation masks for their respective targets. Expanding our validation using these resources would allow for a more robust evaluation. Additionally, as our approach was designed to address zero-shot performance validation, we did not perform any additional training such as fine-tuning. Performance improvements can be expected by using task-specific supervised methods instead of zero-shot. Furthermore, while we used an automated approach to evaluate a large number of organs, there is potential for improved accuracy through manual prompts inputting.

In conclusion, SAM 2 has demonstrated promising zero-shot performance in segmenting certain abdominal organs in CT scans, particularly larger organs with clear boundaries, highlighting its potential for cross-domain generalization in medical imaging. However, further improvements are needed for smaller and less distinct structures. Our study underscores the importance of applying general models to unseen medical images and optimizing input prompts, which together could significantly enhance the accuracy of medical image segmentation.

## Supplementary material

10.2196/72109 Multimedia Appendix 1 Graphs and boxplots displaying mean areas of organs at various levels and comparison of DSCs.

## Abbreviations:

CLAIM: Checklist for Artificial Intelligence in Medical Imaging
CT: computed tomography
DSC: dice similarity coefficient
EKNZ: Ethics Committee Northwest and Central Switzerland
MRI: magnetic resonance imaging
SAM: Segment Anything Model
