# Supplementary material for: Using Segment Anything Model 2 for Zero-Shot 3D Segmentation of Abdominal Organs in Computed Tomography Scans to Adapt Video Tracking Capabilities for 3D Medical Imaging: Algorithm Development and Validation
Source: JMIR AI. 2025 Apr 29;4:e72109. doi: 10.2196/72109 (PMC12231515; doi:10.2196/72109)
Supplement: Multimedia Appendix 1 [file ai-v4-e72109-s001.docx]

**Supplemental Materials**

**
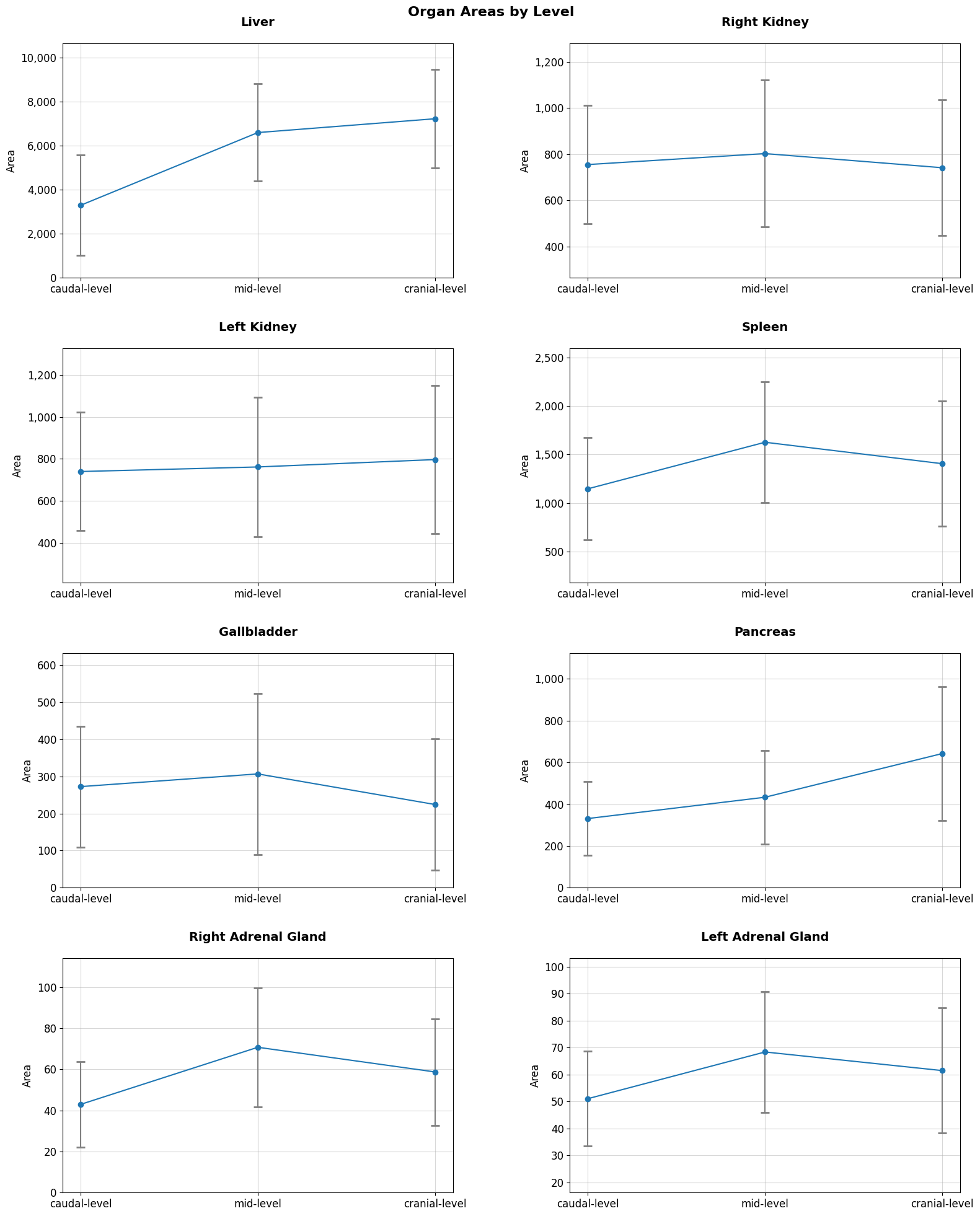
**

Supplemental Figure 1: Comparison of organ areas across different levels. The graph displays the mean areas (in voxel) of organs at the caudal-level, mid-level, and cranial-level. Error bars represent standard deviations.


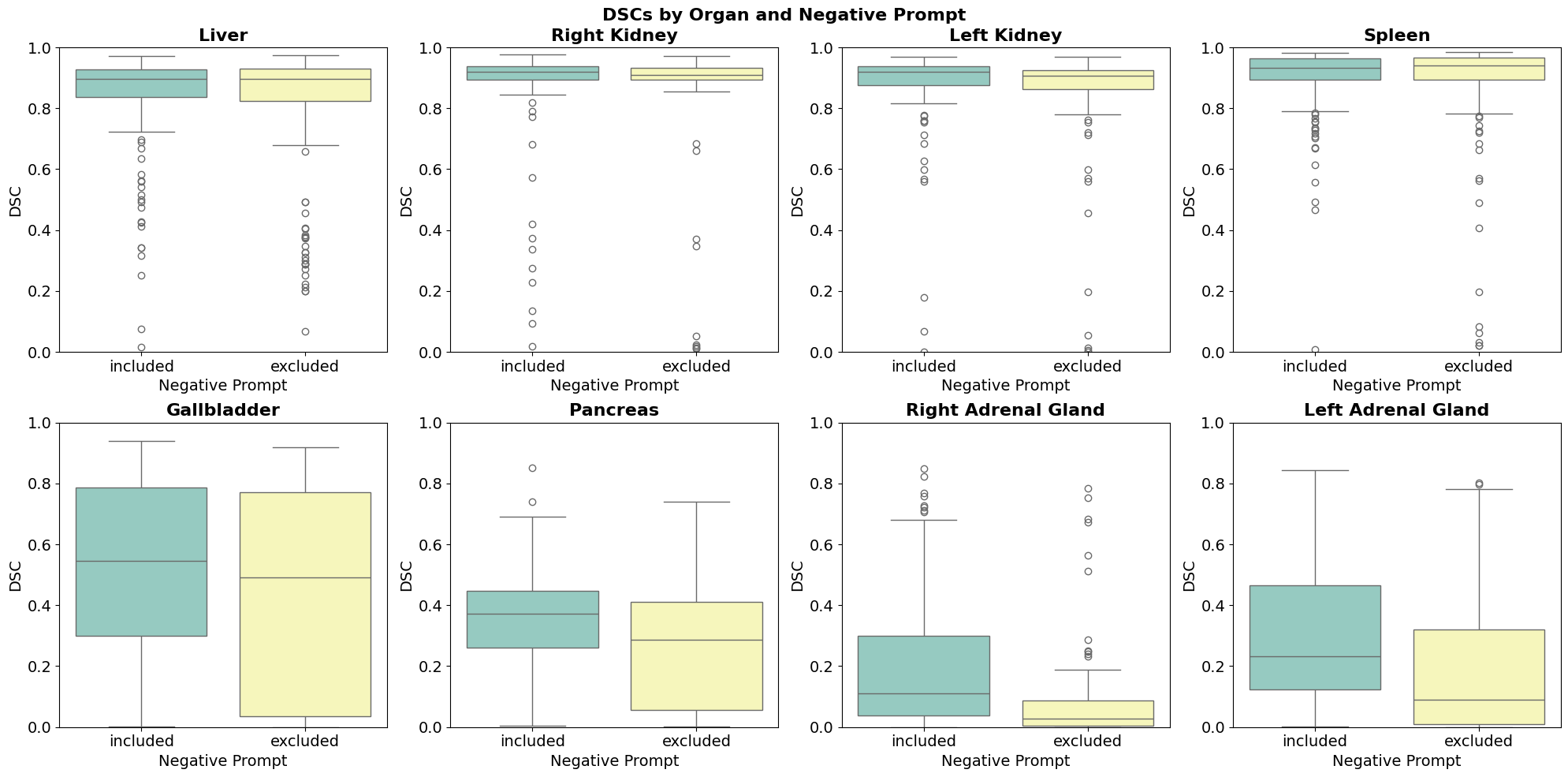


Supplemental Figure 2: Box plots comparing DSCs for eight organs with and without the inclusion of negative prompts. For each organ, DSC values are compared between two conditions: when negative prompts are included (left boxes) and when they are excluded (right boxes). DSC: Dice Similarity Coefficient.
